# Comparison of methods for detecting asymptomatic malaria infections in the China–Myanmar border area

**DOI:** 10.1186/s12936-017-1813-0

**Published:** 2017-04-20

**Authors:** Yonghong Zhao, Yan Zhao, Yanmin Lv, Fei Liu, Qinghui Wang, Peipei Li, Zhenjun Zhao, Yingjie Liu, Liwang Cui, Qi Fan, Yaming Cao

**Affiliations:** 1 Department of Immunology, China Medical University, Shenyang, Liaoning China; 2 Dalian Institute of Biotechnology, Dalian, Liaoning China; 3 Department of Entomology, Pennsylvania State University, University Park, PA USA; 4 Department of Pathogen Biology, China Medical University, Shenyang, Liaoning China

**Keywords:** Malaria, Light microscopy, Nested PCR with DNA, Nested RT-PCR, Capture and ligation probe-PCR, Asymptomatic, Sensitivity, Specificity

## Abstract

**Background:**

Sensitive methods for detecting asymptomatic malaria infections are essential for identifying potential transmission reservoirs and obtaining an accurate assessment of malaria epidemiology in low-endemicity areas aiming to eliminate malaria. PCR techniques to detect parasite nucleic acids (DNA or RNA) are among the most commonly used molecular methods. However, most of these methods are of low throughput and cannot be used for large-scale molecular epidemiological studies. A recently developed capture and ligation probe-PCR (CLIP-PCR) is claimed to have the sensitivity of molecular techniques and the high throughput capacity needed for screening purposes. This study aimed to compare several molecular methods for detecting asymptomatic and submicroscopic *Plasmodium* infections in healthy residents of a malaria-hypoendemic region in Southeast Asia, where malaria elimination is in sight.

**Method:**

This study compared three molecular detection methods side-by-side, namely nested PCR targeting the rRNA genes, nested RT-PCR to detect parasite rRNA, and CLIP-PCR to detect parasite rRNA in 1005 healthy individuals in northeastern Myanmar. For nested PCR and RT-PCR, parasite DNA and total RNA were extracted from ~100 µL of blood, whereas RNA used for CLIP-PCR was from a 3 mm disk of dried blood filter paper. The sensitivity and specificity of these methods were compared with those of conventional light microscopy. In addition, RT-PCR and quantitative RT-PCR (qRT-PCR) targeting the *Pvs25* gene in *Plasmodium vivax* were used to assess gametocyte prevalence in the samples.

**Results:**

Light microscopy detected *Plasmodium* infections in only 1.19% of the residents harbouring the parasites. CLIP-PCR had slightly better performance and detected *Plasmodium* infections in 1.89% of the population. Further improvement was achieved by nested PCR to detect parasite DNA, which detected *P. vivax* and *Plasmodium falciparum* infections in 2.39% of the residents. The nested RT-PCR targeting rRNA, however, detected as many as 187 (18.61%) individuals having *Plasmodium* infections with *P. vivax* being the predominant species (176 *P. vivax,* 5 *P. falciparum* and 6 *P. falciparum/P. vivax* mixed infections). Of the 210 *Plasmodium*-positive samples detected by all molecular methods, 115 were *Pvs25*-positive by qRT-PCR, indicating that a large proportion of asymptomatic individuals were gametocyte carriers.

**Conclusion:**

Nested RT-PCR based on the detection of asexual-stage parasite rRNA was the most sensitive, with a more than sixfold higher sensitivity than the other two molecular methods of parasite detection. CLIP-PCR has an increased throughput, but its sensitivity in this study was much lower than those of other molecular methods, which may be partially due to the smaller amount of RNA input used.

**Electronic supplementary material:**

The online version of this article (doi:10.1186/s12936-017-1813-0) contains supplementary material, which is available to authorized users.

## Background

Malaria is a highly prevalent disease in tropical and subtropical regions, and nearly half of the world’s population is at risk of contracting it [1]. The rapidly shrinking malaria map takes us a step closer to worldwide malaria eradication. Yet, great challenges remain. To achieve elimination and prevent resurgence, surveillance systems must adapt to the changing malaria epidemiology and be able to detect all possible malaria infections in a timely manner. Thus, the accurate identification of all malaria infections, including symptomatic and asymptomatic, has become a vital component of the control and elimination programmes [2]. Asymptomatic malaria infection refers to malarial parasitaemia of any density in the absence of fever or other acute symptoms in individuals who have not received recent antimalarial treatments [3]. Some asymptomatic infections have parasitaemia levels that are detectable by microscopy, whereas others can only be detected by molecular methods and are termed submicroscopic infections. At any given time, the vast majority of individuals with detectable malaria parasitaemia can be categorized as asymptomatic [4], and they are regarded as important reservoirs sustaining malaria transmission [5]. Therefore, low-cost, highly sensitive and specific screening tools would be very useful in the malaria elimination phase [6].

Light microscopy (LM) is the cost-effective, gold standard for detecting symptomatic infections, but it has limitations for the diagnosis of malaria in asymptomatic individuals, especially in low-endemic settings [6–13]. It has been reported that both microscopy and rapid diagnostic tests miss infections when parasite densities are low (<10 parasites/µL) [14]. PCR is the most frequently used molecular method for detecting malaria. Several different target genes have been used, including the 18S ribosomal RNA gene (*18S rRNA*) [15–19], *tRNA* [20], *AMA1* [21], and *cytochrome b* [22, 23], among which the 18S rRNA gene is the most commonly used [11, 24–26]. PCR-based methods include nested PCR with DNA (nD-PCR) [12, 24, 27–31], nested reverse transcriptase PCR (nRT-PCR) [26, 30], quantitative RT-PCR [27, 32–35], and more recently, capture and ligation probe-PCR (CLIP-PCR) [36]. PCR can typically detect 5–10 parasites/µL, while nRT-PCR can detect as few as 22 parasites/mL [26]. In addition, CLIP-PCR has been advocated for use in molecular epidemiological studies because of its higher throughput since samples can be pooled for analysis. It is necessary to compare the benefits of different methods, particularly for asymptomatic malaria.

Whereas most epidemiological surveillance has focused on the evaluation of parasite prevalence in representative populations, gametocyte carriage rarely has been assessed simultaneously. In this study, the prevalence of malaria infections was evaluated in 1005 healthy individuals in villages along the international border between China and Myanmar, where malaria elimination action plans are in place. The sensitivity and specificity of LM and three molecular diagnostic methods for detecting asymptomatic *Plasmodium* infections were compared. In addition, the relationship between parasite species detection using the *Pv18s rRNA* and gametocyte detection using the *Pvs25* gene within individual samples was analysed.

## Methods

### Study area and sample collection

The study site is located in the northeastern Kachin State of Myanmar, along the China-Myanmar border (97.56°E and 24.75°N) [37]. One thousand and five healthy individuals (344 males and 661 females, ages 1–82 years) living in seven villages near the Laiza township were recruited in May (530), July (235) and November (240) of 2015. Finger-prick blood samples (~100 µL) were collected on Whatman 3 M filter paper, air-dried, individually sealed in plastic bags, and stored at −20 °C until use. In addition, 100 µL of finger-prick blood were collected in EDTA tubes, kept on ice, and transferred to a nearby field laboratory on the same day for processing. The study protocol was approved by institutional review boards of the Pennsylvania State University and the local Bureau of Health in Kachin. All participants or legal guardians gave written informed consent before entering the study.

### Malaria diagnosis by LM

Thick and thin blood films stained with Giemsa were prepared and read according to the World Health Organization standard operating procedure in basic malaria microscopy with an oil immersion lens (100×) by two microscopists who had at least five years of experience. Each slide was examined for at least 100 good fields by each microscopist. For positive slides, parasite density was quantified in 500 white blood cells (WBCs) on thick blood films assuming that 1 µL of blood contains 8000 WBCs [38]. Thin films in the positive slides were further examined to identify the parasite species. For samples with discrepant results by the two microscopists, a third senior microscopist provided additional evaluation to reconcile the divergence.

### Nucleic acid extraction and cDNA synthesis

Total RNA and genomic DNA were extracted from peripheral blood samples with Trizol (Invitrogen, Carlsbad, CA, USA) according to the manufacturer’s instructions. Briefly, 0.1 mL of blood sample in a 1.5 mL tube was mixed with 1 mL of Trizol and incubated at room temperature for 5 min. Two hundred µL of chloroform was added and mixed vigorously by hand for 15 s. Phase separation was done by centrifugation at 12,000×*g* for 15 min at 4 °C. The aqueous phase was transferred to a fresh 1.5 mL tube, where 10 µg of the carrier GlycoBlue Coprecipitant (Invitrogen) and 0.5 mL of 100% isopropanol were added to precipitate total RNA. The RNA pellet was washed with 1 mL of 75% ethanol twice, air-dried, dissolved in 30 µL RNase-free water, and stored at −70 °C. Genomic DNA was isolated from the interphase and phenol phase following the protocol for DNA isolation. Genomic DNA was dissolved in 30 µL of 8 mM HEPES buffer at pH 7.0–8.0 and stored at −20 °C. One microgram of each total RNA sample was directly used as the template for nested-PCR with primers for the *Plasmodium* 18S rRNA gene to verify that isolated total RNA had no genomic DNA contamination.

cDNA was synthesized from 1 µg of each RNA sample (~1/6 to 1/4 of total RNA) using the Takara RNA PCR kit (AMV) version 3.0 (Takara, Japan) in a total volume of 20 µL consisting of 1 µg RNA, 4 µL 5× reverse transcriptase buffer, 2 µL dNTP mix (10 mM each),1 µL primer of Random 9mer mixed with oligo dT-adaptor primer, 20 U (0.5 µL) RNase inhibitor, 10 U (2 µL) AMV reverse transcriptase, and RNase-free water to 20 µL.

### PCR detection targeting the *18S rRNA*

#### Nested PCR with genomic DNA (nD-PCR) or cDNA (nRT-PCR)

Modified nested PCR (nD-PCR) was performed as previously described based on the 18S rRNA gene [12, 15, 31, 39–41]. *Plasmodium* genus- and species-specific primers for *P. falciparum*, *P. vivax*, *Plasmodium malariae*, and *Plasmodium ovale*, and expected sizes of PCR fragments and PCR reaction conditions are shown in Additional file 1. Primary PCR reactions were performed in 25 µL containing 14 µL distilled H_2_O, 1.0 µL each of rPLU5 and rPLU1 primers (10 µmol/L), 2.5 µL 10 × buffer, 2 µL dNTP mixture (2.5 mM), 0.5 µL rTaq (2.5 U), and 4 µL of genomic DNA. Nested PCR was performed used 2 µL of the primary PCR product as a template and species-specific primers for the four human malaria species in separate reaction tubes. PCR products were separated in 1.2% agarose gels. For PCR assessment, one positive control (from a symptomatic *P. vivax* case) and one negative control (sterile water) were used in a blind test for analysis. nRT-PCR was performed similarly as for nD-PCR using 1 µL cDNA template in the primary PCR. Three clinical *P. vivax* samples with an average density of 3000, 3800 and 4400 parasites/µL blood, respectively, were used to define the limits of detection (LOD) for nD-PCR and nRT-PCR. The average parasite density of each sample was determined by two microscopists, who counted parasites per 500 WBCs in thick smears assuming 8000 WBCs/µL blood. All three samples were first diluted to the same parasite density using the same whole blood from a healthy person, and then subjected to fourfold serial dilutions (2000–0.488 parasites/µL) and threefold serial dilutions (5.6–0.0026 parasites/µL) for nD-PCR and nRT-PCR, respectively. For each dilution and nucleic acid extraction, nD-PCR or nRT-PCR was performed in triplicates, and the lowest parasite density at which all three PCR replicates were positive was considered the LOD.

### Capture and ligation probe-PCR (CLIP-PCR)

For CLIP-PCR, a 3-mm circle of dried blood spot on 3 M Whatman filter paper was punched out and lysed with 100 µL lysis mixture (Diacurate, Paris, France), 191 µL water, 3 µL mixed capture and detection probe for the *Plasmodium* genus, and 6 µL proteinase K (50 g/L) at 56 °C for 30 min with vigorous shaking. Pooled sample spots were lysed in the same manner. Lysates were then transferred at 100 µL per well to a 96-well capture plate (Diacurate). After incubation at 55 °C for 3 h, each well was washed three times with 150 µL wash buffer and incubated with 50 µL ligation mix at 37 °C for 30 min. The plate was then washed again and used for qPCR with 25 µL/well of PCR mixture containing 1× SYBR^®^ Premix Ex (Takara) and 100 nmol/L primers. Amplification and detection were performed on an ABI 7500 apparatus (Applied Biosystems, Foster City, CA, USA) under the following conditions: 30 s at 95 °C, 45 cycles of 5 s at 95 °C, and 20 s at 60 °C. The melting curve was prepared from 65 °C to 90 °C using a default setting. The standard curve was made by threefold serial dilutions (8–0.004 parasites/µL) of a lysate of cultured *P. falciparum* 3D7 strain diluted with a parasite-negative whole blood lysate. For CLIP-PCR, the sample was considered positive if the fluorescent signal increased within 29 cycles and the melting curve was the same as that of the positive control [36].

### RT-PCR detection targeting the *Pvs25* gene

Two RT-PCR methods were used to detect *P. vivax* gametocytes in samples. For detection of *P. vivax* gametocytes in all 1005 samples, the 645 bp full-length *Pvs25* gene was amplified using *Pvs25*-specific primers under specified reaction conditions (Additional file 1: Table S1) [42]. The PCR reaction contained 2 µL of 10× KOD-Plus-Neo buffer, 2 µL of 2 mM dNTPs, 0.8 µL of 25 mM MgSO_4_, 0.5 µL of 10 µM of pvs25_fw and pvs25_rev primers, 0.5 U of KOD Plus-Neo DNA polymerase (Toyobo, Osaka, Japan), and 1.0 µL cDNA in a final volume of 20 µL. PCR products were separated on 1.2% agarose gels. Three *P. vivax* gametocyte-positive samples with average gametocyte densities of 240, 208, and 320 gametocytes/µL blood, respectively, were used to define the LOD of the Pvs25 RT-PCR. Similarly, threefold serial dilutions of the three *P. vivax* gametocyte-positive samples (all diluted to 5.6–0.0026 gametocytes/µL) were used for RT-PCR, and the lowest gametocyte density at which all three PCR replicates were positive was considered the LOD.

The subset of samples that were positive for *P. vivax* parasites was further analysed by the TaqMan probe-based quantitative RT-PCR (qRT-PCR) targeting the *Pvs25* transcript [43]. Sequences of primers as well as the FAM-BHQ1-labeled probes for *Pvs25* (GenBank Accession No. XM_001608410), expected size of the PCR fragment and PCR conditions are shown in Additional file 1. The PCR reaction consisted of 10 µL of 10× primer Extaq buffer, 0.2 µL of 50× Rox Reference Dye II, 0.8 µL of pvs25_probe, 0.4 µL of 10 µM of pvs25_fw and pvs25_rev primers, 1.0 µL cDNA, and sterile water to 20 µL (Takara). A 115 bp fragment of *Pvs25* was amplified with primers pvs25_fw and pvs25_rev from the cDNA of a symptomatic *P. vivax* gametocyte-positive case, cloned into the pMD-18T vector (Takara), sequenced, and used as the positive control plasmid. Tenfold dilutions of plasmid DNA (3 × 10^9^ to 3 × 10^−1^ copies/µL) were made in triplicates to calculate a standard curve. The amplification efficiency (E) was calculated using the slopes of the standard curves (E = 10^(−1/Slope)^ − 1). The LOD was measured using the threefold serial dilutions of the same three *P. vivax* gametocyte-positive samples (all diluted to 9–0.001 gametocytes/µL). qRT-PCR was carried out on an ABI 7500 apparatus (Applied Biosystems, Foster City, CA, USA) and analysed with 7500 Fast Software v2.3. To identify gametocyte-positive samples, the C_t_ values of standard curves obtained from assay-specific plasmids were routinely included in each 96-well plate.

### Statistical analysis

Pair-wise comparison among the proportions of positive detections for the different methods was made by the McNemar’s exact test. Sensitivity and specificity were calculated from the numbers of true/false positives and negatives when each of the methods was considered to be the reference method by statistical analysis software SPSS and data combined using Microsoft Excel 2010 for Windows.

## Results

### Prevalence of *Plasmodium* infections detected by different methods

**Fig. 1:**
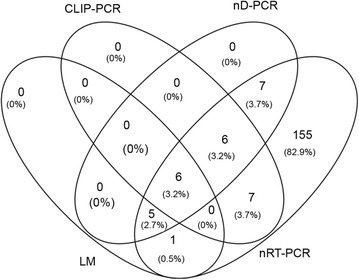
Venn diagram showing the overlap in the number (percent) of individuals with asymptomatic *Plasmodium* infections in a population of 1005 as detected by light microscopy (LM) and one of the three molecular methods targeting parasite 18S rRNA genes (*nD-PCR* nested PCR using genomic DNA, *nRT-PCR* nested RT-PCR using parasite total RNA, *CLIP-PCR* capture and ligation probe-PCR)

LM and three molecular detection methods targeting the asexual parasite 18S rRNA genes were compared side by side using 1005 blood samples collected from healthy residents in villages of a malaria-endemic area from northeastern Myanmar. LM only detected a total of 12 (1.19%) *P. vivax* infections. Both nD-PCR and nRT-PCR methods detected *Plasmodium* infections based on the expected sizes of the PCR fragments on agarose gels, while CLIP-PCR was based on the melting curves of the positive controls (Additional file 2). The method based on CLIP-PCR detected only a few additional infections compared to LM (19 infections, 1.89%) (Table 1). In comparison, nD-PCR detected 24 (2.39%) malaria infections, including 23 *P. vivax* and 1 mixed *P. falciparum/P. vivax* infections (Table 1). The RNA-based detection method nRT-PCR was the most sensitive, and detected 187 (18.61%) malaria infections, including 176 *P. vivax,* 5 *P. falciparum*, and 6 mixed *P. falciparum/P. vivax* infections (Table 1). No *P. malariae* or *P. ovale* infections were observed by any of the methods. Statistical analysis showed that all pair-wise comparisons of the detection rates of the four methods were significantly different (p < 0.05) (Table 1). Of the total 182 *P. vivax* and *P. falciparum/P. vivax* positive samples by nRT-PCR, 32 samples were *P. vivax*-positive by at least one of the other detection methods (LM, CLIP-PCR, or nD-PCR) (Fig. 1).

**Table 1:** Performance of different detection methods on asymptomatic malaria infections

	LM	nD-PCR	nRT-PCR	CLIP-PCR
*P. vivax*	12	23	176	–
*P. falciparum*	0	0	5	–
*P. falciparum* and *P. vivax*	0	1	6	–
*Plasmodium* spp.	12	24	187	19
Total	1005	1005	1005	1005
Positive rate (%)	1.19	2.39	18.61	1.89

*LM* light microscopy, *nD-PCR* nested PCR using genomic DNA, *nRT-PCR* nested RT-PCR using parasite total RNA, *CLIP-PCR* capture and ligation probe-PCR

### Sensitivity and specificity of different methods of *Plasmodium* detection

Comparisons of sensitivity and specificity of the malaria detection methods are presented in Table 2. LM traditionally has been considered the ‘gold standard’, but its low sensitivity would lead to biased estimates for ‘false positives’ if it is used as the only reference method. Thus, comparisons were made with each method being considered as the reference. When the least sensitive LM method served as the reference, nD-PCR and nRT-PCR both showed high sensitivity (>90%), whereas CLIP-PCR only had ~50% sensitivity. When the commonly used nD-PCR was used as the reference, CLIP-PCR again showed modest sensitivity of ~50%, similar to that of LM. Compared to the most sensitive method of nRT-PCR, all other methods showed low sensitivity (6.4–10.2%). For all comparisons, the specificity was high (>95%) except for that of the nRT-PCR method (~83%), suggesting that nRT-PCR detected more infections than other methods, which can be expected given the large difference between the LOD of nRT-PCR and those of other methods, while the positives detected by the rest of the methods had significant overlaps. It is noteworthy that the CLIP-PCR method, despite increased throughput, had only modest sensitivity compared to LM and nD-PCR.

**Table 2:** Comparison of sensitivity and specificity of *Plasmodium* detection methods

Reference	Pos	Neg	Pos	Neg	Pos	Neg	Total
	CLIP-PCR		nD-PCR		nRT-PCR		
LM							
Pos	6	6	11	1	12	0	12
Neg	13	980	13	980	175	818	993
Total	19	986	24	981	187	818	1005
Sensitivity (95% CI)	50.0% (46.9–53.0%)		91.7% (90–93.4%)		100% (100–100%)		
Specificity (95% CI)	98.7% (92.3–100%)		98.7% (92.3–100%)		82.4% (60.8–100%)		

Reference	LM		nD-PCR		nRT-PCR		Total
CLIP-PCR							
Pos	6	13	12	7	19	0	19
Neg	6	980	12	974	168	818	986
Total	12	993	24	981	187	818	1005
Sensitivity (95% CI)	31.6% (28.7–34.5%)		63.2% (60.1–66.2%)		100% (100–100%)		
Specificity (95% CI)	99.4% (95.9–100%)		98.8% (93.9–100%)		83.0% (66.0–99.9%)		

Reference	CLIP-PCR		LM		nRT-PCR		Total
nD-PCR							
Pos	12	12	11	13	24	0	24
Neg	7	974	1	980	163	818	981
Total	19	986	12	993	187	818	1005
Sensitivity (95% CI)	50.0% (46.9–53.1%)		45.8% (42.7–49.0%)		100% (100–100%)		
Specificity (95% CI)	99.3% (95.9–100%)		99.9% (98.6–100%)		83.38% (68.5–98.3%)		

Reference	CLIP-PCR		nD-PCR		LM		Total
nRT-PCR							
Pos	19	168	24	163	12	175	187
Neg	0	818	0	818	0	818	818
Total	19	986	24	981	12	993	1005
Sensitivity (95% CI)	10.2% (8.1–12.2%)		12.8% (10.5–15.1%)		6.4% (4.8–8.1%)		
Specificity (95% CI)	100% (100–100%)		100% (100–100%)		100% (100–100%)		

The aforementioned sensitivity of different methods is consistent with their LODs. For nD-PCR, the LODs of all three samples were determined to be ~2 *P. vivax* parasites/µL. A 200-fold increase in sensitivity was observed for nRT-PCR, which had a LOD of 0.01 *P. vivax* parasites/µL for the three parasite samples. For CLIP-PCR using the 3D7 parasite lysate, the LOD was similarly low at 0.01 parasites/µL (Additional file 3). However, its LOD for *P. vivax* was not determined.

### Prevalence of sexual stages of *Plasmodium vivax*

Given that the blood samples from the surveys contained mostly *P. vivax* infections, the presence of *P. vivax* gametocytes was further analysed. First, all 1005 blood samples were detected by RT-PCR targeting the *Pvs25* gene, which detected a total of 61 *P. vivax* gametocyte-positive infections based on the 645 bp PCR product (Table 3). Of the 61 *P. vivax* gametocyte-positive infections, 33 were *P. vivax* 18S rRNA-positive cases and 28 were *P. vivax*-negative infections by all 18S rRNA gene-based molecular methods (Table 3). These latter samples may represent *P. vivax* infections with the presence of gametocytes but absence of asexual stages. When both 18S rRNA-based nRT-PCR and Pvs25-based RT-PCR were considered, the number of *P. vivax*-positive samples increased to 210, which also included six *P. falciparum/P. vivax* mixed infections.

**Table 3:** *P. vivax* gametocyte detection *Pvs25* RT-PCR and parasite detection by other methods

RT-PCR (Pvs25)	CLIP-PCR		LM		nRT-PCR		nD-PCR		Total
	Positive	Negative	Positive	Negative	Positive	Negative	Positive	Negative	
Positive	7	54	7	54	33	28	9	52	61
Negative	12	932	5	939	149	795	15	929	944
Total	19	986	12	993	182	823	24	981	1005

The presence of gametocytes in the 210 *P. vivax*-positive blood samples was further evaluated using a more sensitive qRT-PCR based on *Pvs25* mRNA. Based on the standard curve from the serially diluted Pvs25 plasmid (Additional file 4), the C_t_ value of <33 was used as the threshold for *P. vivax* gametocyte positivity. Of the 210 *P. vivax* samples, 115 were determined to be gametocyte-positive by qRT-PCR, and this confirmed the 61 *Pvs25*-positive samples by RT-PCR as gametocyte-positive (Table 4). Among them, 7/12 (58%) LM-positive, 11/19 (58%) CLIP-PCR-positive, 11/24 (46%) nD-PCR-positive, and 87/182 (47%) nRT-PCR-positive *P. vivax* infections harboured gametocytes detected by the *Pvs25*-based qRT-PCR. Whereas all gametocyte-positive samples detected by LM contained asexual-stage parasites, 28 gametocyte-positive samples detected by RT-PCR and qRT-PCR were not positive by the 18S rRNA-based methods. For the qRT-PCR, the LOD was estimated to be 3 copies/µL of plasmid (Additional file 4), while the estimate of LOD from three *P. vivax* gametocyte-positive samples was ~0.004 gametocyte/µL. The LOD for RT-PCR was 0.02 parasites/µL.

**Table 4:** Gametocyte detection by *Pvs25* mRNA based detection (RT-PCR and qRT-PCR) in 210 *P. vivax*-positive samples and their overlaps with parasite detection by other methods

qRT-PCR (*Pvs25)*	LM		CLIP-PCR		nD-PCR		nRT-PCR		RT-PCR (*Pvs25*)		Total
	Positive	Negative	Positive	Negative	Positive	Negative	Positive	Negative	Positive	Negative	
Positive	7	108	11	104	11	104	87	28	61	54	115
Negative	5	90	8	87	13	82	95	0	0	95	95
Total	12	198	19	191	24	186	182	28	61	149	210
Gametocyte carriers (%)	7/12 (58.33%)		11/19 (57.89%)		11/24 (45.83%)		87/182 (47.80%)		61/61 (100%)		115/210 (54.76%)

RT-PCR (*Pvs25*)	LM		CLIP-PCR		nD-PCR		nRT-PCR		qRT-PCR (*Pvs25*)		
Positive	7	54	7	54	9	52	33	28	61	0	61
Negative	5	144	12	137	15	134	149	0	54	95	149
Total	12	198	19	191	24	186	182	28	115	95	210
Gametocyte carriers (%)	7/12 (58.33%)		7/19 (36.84%)		9//24 (37.50%)		33/182 (18.13%)		61/115 (53.04%)		61/210 (29.05%)

## Discussion

Asymptomatic infections of malaria play an important role in sustaining transmission and present an obstacle to malaria elimination in low-endemicity regions [5]. Accurate knowledge of malaria epidemiology and transmission dynamics of the parasites in pre-elimination regions is critical for implementing effective and targeted control measures such as detect-and-treat and mass drug administration strategies. Low-cost, highly sensitive and specific screening tools for malaria are required for this purpose [44]. Molecular detection methods, though not suitable for field operations on a large scale, are normally applied in order to obtain representative assessment of the malaria situation in a subpopulation of an area. With the ease and speed of detection, PCR is a commonly used molecular tool and the detection limit is generally 50–100 times lower than those of LM and RDT [24, 45–47]. CLIP-PCR recently has been developed with a claimed level of sensitivity as low as 0.01 parasites/µL and a much increased throughput that might be suitable for active screening of malaria parasites in low-transmission settings [36]. This study compared the sensitivity and specificity of both DNA- and RNA-based methods for detecting *Plasmodium* infections during cross-sectional surveys in an area of the Greater Mekong Subregion, which aims to eliminate malaria by 2030. This study identified that the two RNA-based detection methods, one for detecting *Plasmodium* 18S rRNA in asexual stages and the other for detecting the *Pvs25* in gametocytes, were the most sensitive and detected a major proportion of the infections as submicroscopic.

WHO recommend LM as the ‘gold standard’ for symptomatic malaria, but its performance for detecting asymptomatic infections, especially under low endemic settings, is generally poor. In this survey, LM only detected 1.19% of the study population carrying asymptomatic *P. vivax* infections, which is consistent with prior reports of a threshold for LM of around 10 parasites/µL for a research setting [48] and 50–100 parasites/µL for outside a research setting [49]. Because of low parasite densities with the asymptomatic infections, LM is time-consuming and has much lower sensitivity than molecular methods. Thus, it is not favored for screening for asymptomatic infections in low-endemic settings like the present one.

Species-specific nD-PCR is a frequently used method in molecular epidemiological studies since parasite DNA can easily be preserved on filter papers, and cheap DNA-binding agents such as Chelex can be used for DNA extraction [50]. This method, in our hands, had a parasite detection limit of less than 2 parasites/µL, similar to an earlier report [6], and the number of infections detected was more than double that detected using LM. In comparison, nRT-PCR, based on the detection of asexual stage rRNA with a LOD of 0.01 parasites/µL blood, detected 18.61% of the study population as *Plasmodium* carriers. The presence of ~3500 18S rRNA transcripts in a single asexual parasite circulating in peripheral blood largely explains the superior sensitivity of nRT-PCR [26, 51]. Furthermore, this study extracted RNA directly from freshly collected blood samples, which may have improved the efficiency of RNA extraction.

This study specifically assessed the detection efficiency of the recently developed CLIP-PCR. Though this method had a LOD of as low as 0.01 parasites/µL of *P. falciparum,* it performed only slightly better than LM and detected 1.89% of the population carrying *Plasmodium* infections. First, the increased throughput means that significant pooling of the samples was used. With 500 tests in a 96-well plate, CLIP-PCR incurs significant pooling of the samples and dilution of the targets [36]. Second, this method used parasite RNA preserved on filter papers without the addition of any stabilizers, thus target degradation may also have accounted for the lower detection sensitivity. Third, the LOD was determined for *P. falciparum*, which might be different for *P. vivax*. Furthermore, the inferior performance of CLIP-PCR may be due to lower number of parasites used. For nD-PCR, RT-PCR, and nRT-PCR, the nucleic acids were extracted from 100 µL of whole blood. For nD-PCR, the amount of DNA used per reaction (4/30 µL of total DNA) corresponded to ~13 µL of whole blood, while the amount of RNA used for RT-PCR and nRT-PCR corresponded to ~1 µL of whole blood. In comparison, the CLIP-PCR used a 3-mm punch of dried filter paper, which is likely equivalent to <10 µL of whole blood. Nevertheless, CLIP-PCR demands further testing and improvement if future uses in molecular epidemiological studies in low endemic settings are considered. However, the lack of transparency on the design, and unavailable information about the sequences of the capture or detection probes and the kit components (of the assay lysis mixture, wash buffers, or ligation mix), hinder wide applications of this method [52].

With the predominant status of *P. vivax* infections in the study area, the presence of gametocytes also was evaluated with two RT-PCR methods targeting the *Pvs25* transcripts. RT-PCR for *Pvs25* detected gametocyte carriage in 6% (61/1005) of the study population, further increasing the *P. vivax* infection rate from 18% to 20%. Analysis of the *P. vivax*-positive samples from other methods by qRT-PCR targeting *Pvs25* transcripts revealed 115 of them as gametocyte-positive. Interestingly, 101 of the 182 *P. vivax*-positive samples detected by nRT-PCR targeting the 18S rRNA were gametocyte-positive, whereas 28 samples were only *Pvs25* positive. Negativity by nRT-PCR in a gametocyte-positive sample could be explained by the presence of significantly higher numbers of gametocytes (*Pvs25* transcripts) than the asexual forms [43], which may have attributed to host conditions (including pH, drug, immunity, anaemia) that stimulate gametocyte formation and decrease asexual parasites [10, 53]. Nevertheless, the relatively high rates of gametocyte carriage suggest that a large proportion of the asymptomatic and submicroscopic infections may serve as important reservoirs of continued malaria transmission in this area of low endemicity.

This study identified the nRT-PCR method targeting the 18S rRNA as an extremely sensitive, robust, and scalable procedure for molecular surveillance. A considerable overlap of detected infections with Pvs25-based method further indicates the validity of this method. The sensitivity (LOD of 10 parasites/mL) is similar to the high-volume qPCR method (>20 parasites/mL) that uses venous blood [25], which is logistically difficult to conduct in large epidemiological studies. The availability of improved methods for conserving nucleic acids before processing will guarantee detection of malaria prevalence even in remote regions [19, 25, 26].

## Conclusions

A survey method capable of identifying virtually every individual infected with *Plasmodium* parasites will be crucial in the malaria elimination phase. Comparison of LM with three molecular detection methods for parasite 18S rRNA genes and RT-PCR for the Pvs25 gene was conducted with samples from cross-sectional surveys in a malaria-hypoendemic area, which demonstrated the superior efficiency of nRT-PCR and qRT-PCR as surveillance tools to detect asexual parasite (18S rRNA) and gametocyte (*Pvs25*) infections, respectively. CLIP-PCR, though with the highest throughput among the molecular detection methods used, had a much lower sensitivity, which might be due to differences in the amount of the starting clinical samples used. This study showed a large proportion of *Plasmodium*-positive individuals as gametocyte carriers, highlighting the importance of transmission-interruption strategies for malaria elimination.

## Additional files

**Additional file 1.** PCR primer sequences and reaction conditions.

**Additional file 2.** Nested PCR targeting 18S rRNA with parasite genomic DNA (nD-PCR) and cDNA (nRT-PCR). A. PV-18S rRNA (419 bp) by nD-PCR. B. PF-18S rRNA (205 bp) by nD-PCR. C. PF-18S rRNA (205 bp) by nRT-PCR. D. PV-18S rRNA (419 bp) by nRT-PCR. E. CLIP-PCR positive sample definedby normal “S” amplification, the dissolution curve has a single peak, and the product Tm is the same as that in positive control (with <0.5 °C difference). PCR products were separated on 1.2% agarose gels (A-D). M, molecular markers in bp. N = negative control, P = positive control.

**Additional file 3.** Limits of detection (LOD) of different detection methods - nD-PCR, nRT-PCR and CLIP-PCR. A. LOD based on *P. vivax* 18S rRNA gene by nD-PCR. Four-fold serial dilutions of the parasites from 2000 to 0.488 parasites/μL were used. Lanes 1-3, 4-6, 7-9, 10-12, 13-15, 16-18 and 19-21 correspond to parasite density of 2000, 500, 125, 31.25, 7.81, 1.95, 0.49 parasites/μL, respectively. B. LOD based on *P. vivax* 18S rRNA by nRT-PCR. Three-fold serial dilutions of the parasites from 5.6 to 0.0026 parasites/μL were used. Lanes 1-3, 4-6, 7-9, 10-12, 13-15, 16-18, 19-21 and 22-24 represent 5.6, 1.87, 0.533, 0.178, 0.059, 0.02, 0.0078 and 0.0026 parasites/μL, respectively. C. LOD of CLIP-PCR. Three-fold serial dilutions of *P. falciparum* 3D7 were used (8 – 0.004 parasites/μL). Eff% is amplification efficiency. Ct values from duplicate tests were plotted against parasite densities.

**Additional file 4.** Limits of detection (LOD) of RT-PCR and qRT-PCR for gametocyte detection. A. LOD based on *P. vivax* Pvs25 rRNA gene by RT-PCR. Three-fold serial dilutions of the gametocytes (5.6 – 0.0026 gametocytes/μL) were used. Lanes 1-3, 4-6, 7-9, 10-12, 13-15, 16-18, 19-21 and 22-24 correspond to gametocyte density of 5.6, 1.87, 0.533, 0.178, 0.059, 0.02, 0.0078 and 0.0026 gametocytes/μL, respectively. B. LOD of qRT- PCR using plasmid DNA. Ten-fold serial dilutions of *P. vivax* Pvs25 plasmid were used (3 ×10^9^ – 0.3 copies/μL). Eff% is the amplification efficiency. Ct values from duplicate tests were plotted against parasite densities. C. LOD of qRT-PCR using *P. vivax* gametocytes. Three-fold serial dilutions of *P. vivax* gametocytes (9 – 0.001 gametocytes/μL) in three samples were used. Eff% is the amplification efficiency. Ct values from duplicate tests were plotted against parasite densities.

## Abbreviations

PCR: polymerase chain reaction
LM: light microscopy
nD-PCR: nested PCR with DNA
nRT-PCR: nested reverse transcription PCR
CLIP-PCR: capture and ligation probe-PCR
RT-PCR: reverse transcriptase PCR
qRT-PCR: quantitative real-time reverse transcriptase PCR

## References

[1] WHO (2016). World malaria report 2016.

[2] Al-Harti SA (2016). Assessment of three blood genomic-DNA Preparation methods for malaria molecular diagnosis. J Egypt Soc Parasitol.

[3] Lindblade KA, Steinhardt L, Samuels A, Kachur SP, Slutsker L (2013). The silent threat: asymptomatic parasitemia and malaria transmission. Expert Rev Anti Infect Ther..

[4] Chen I, Clarke SE, Gosling R, Hamainza B, Killeen G, Magill A (2016). “Asymptomatic” malaria: a chronic and debilitating infection that should be treated. PLoS Med.

[5] Peto TJ, Tripura R, Lee SJ, Althaus T, Dunachie S, Nguon C (2016). Association between subclinical malaria infection and inflammatory host response in a pre-elimination setting. PLoS ONE.

[6] Bell D, Fleurent AE, Hegg MC, Boomgard JD, McConnico CC (2016). Development of new malaria diagnostics: matching performance and need. Malar J.

[7] Elbadry MA, Al-Khedery B, Tagliamonte MS, Yowell CA, Raccurt CP (2015). High prevalence of asymptomatic malaria infections: a cross-sectional study in rural areas in six departments in Haiti. Malar J.

[8] Ogunniyi A, Dairo MD, Dada-Adegbola H, Ajayi IO, Olayinka A, Oyibo WA (2016). Cost-effectiveness and validity assessment of cyscope microscope, quantitative buffy coat microscope, and rapid diagnostic kit for malaria diagnosis among clinic attendees in Ibadan, Nigeria. Malar Res Treat.

[9] Xu G, Nolder D, Reboud J, Oguike MC, van Schalkwyk DA, Sutherland CJ (2016). Paper-origami-based multiplexed malaria diagnostics from whole blood. Angew Chem Int Ed Engl.

[10] Bousema T, Drakeley C (2011). Epidemiology and infectivity of *Plasmodium falciparum* and *Plasmodium vivax* gametocytes in relation to malaria control and elimination. Clin Microbiol Rev.

[11] Kasetsirikul S, Buranapong J, Srituravanich W, Kaewthamasorn M, Pimpin A (2016). The development of malaria diagnostic techniques: a review of the approaches with focus on dielectrophoretic and magnetophoretic methods. Malar J.

[12] Li P, Zhao Z, Wang Y, Xing H, Parker DM, Yang Z (2014). Nested PCR detection of malaria directly using blood filter paper samples from epidemiological surveys. Malar J.

[13] Ali IM, Bigoga JD, Forsah DA, Cho-Ngwa F, Tchinda V, Moor VA (2016). Field evaluation of the 22 rapid diagnostic tests for community management of malaria with artemisinin combination therapy in Cameroon. Malar J.

[14] Harris I, Sharrock WW, Bain LM, Gray KA, Bobogare A, Boaz L (2010). A large proportion of asymptomatic Plasmodium infections with low and sub-microscopic parasite densities in the low transmission setting of Temotu Province, Solomon Islands: challenges for malaria diagnostics in an elimination setting. Malar J.

[15] Snounou G, Viriyakosol S, Jarra W, Thaithong S, Brown KN (1993). Identification of the four human malaria parasite species in field samples by the polymerase chain reaction and detection of a high prevalence of mixed infections. Mol Biochem Parasitol.

[16] Imoukhuede EB, Andrews L, Milligan P, Berthoud T, Bojang K, Nwakanma D, Ismaili J (2007). Low-level malaria infections detected by a sensitive polymerase chain reaction assay and use of this technique in the evaluation of malaria vaccines in an endemic area. Am J Trop Med Hyg.

[17] Okell LC, Ghani AC, Lyons E, Drakeley CJ (2009). Submicroscopic infection in *Plasmodium falciparum*-endemic populations: a systematic review and meta-analysis. J Infect Dis.

[18] Hermsen CC, Telgt DS, Linders EH, van de Locht LA, Eling WM, Mensink EJ (2001). Detection of *Plasmodium falciparum* malaria parasites in vivo by real-time quantitative PCR. Mol Biochem Parasitol.

[19] Kamau E, Tolbert LS, Kortepeter L, Pratt M, Nyakoe N, Muringo L (2011). Development of a highly sensitive genus-specific quantitative reverse transcriptase real-time PCR assay for detection and quantitation of Plasmodium by amplifying RNA and DNA of the 18S rRNA genes. J Clin Microbiol.

[20] Beshir KB, Hallett RL, Eziefula AC, Bailey R, Watson J, Wright SG (2010). Measuring the efficacy of anti-malarial drugs in vivo: quantitative PCR measurement of parasite clearance. Malar J.

[21] Garg S, Alam MT, Das MK, Dev V, Kumar A, Dash AP (2007). Sequence diversity and natural selection at domain I of the apical membrane antigen 1 among Indian *Plasmodium falciparum* populations. Malar J.

[22] Hwang J, Jaroensuk J, Leimanis ML, Russell B, McGready R, Day N (2012). Long-term storage limits PCR-based analyses of malaria parasites in archival dried blood spots. Malar J.

[23] Farrugia C, Cabaret O, Botterel F, Bories C, Foulet F, Costa JM (2011). Cytochrome b gene quantitative PCR for diagnosing *Plasmodium falciparum* infection in travelers. J Clin Microbiol.

[24] Yin JH, Yan H, Huang F, Li M, Xiao HH, Zhou SS (2015). Establishing a China malaria diagnosis reference laboratory network for malaria elimination. Malar J.

[25] Imwong M, Hanchana S, Malleret B, Renia L, Day NP, Dondorp A (2014). High-throughput ultrasensitive molecular techniques for quantifying low-density malaria parasitemias. J Clin Microbiol.

[26] Adams M, Joshi SN, Mbambo G, Mu AZ, Roemmich SM, Shrestha B (2015). An ultrasensitive reverse transcription polymerase chain reaction assay to detect asymptomatic low-density *Plasmodium falciparum* and *Plasmodium vivax* infections in small volume blood samples. Malar J.

[27] Doctor SM, Liu Y, Anderson OG, Whitesell AN, Mwandagalirwa MK, Muwonga J (2016). Low prevalence of *Plasmodium malariae* and *Plasmodium ovale* mono-infections among children in the Democratic Republic of the Congo: a population-based, cross-sectional study. Malar J.

[28] Papa Mze N, Ahouidi AD, Diedhiou CK, Silai R, Diallo M, Ndiaye D (2016). Distribution of Plasmodium species on the island of Grande Comore on the basis of DNA extracted from rapid diagnostic tests. Parasite.

[29] Mapua MI, Petrzelkova KJ, Burgunder J, Dadakova E, Brozova K, Hrazdilova K (2016). A comparative molecular survey of malaria prevalence among Eastern chimpanzee populations in Issa Valley (Tanzania) and Kalinzu (Uganda). Malar J.

[30] Kuamsab N, Putaporntip C, Pattanawong U, Jongwutiwes S (2012). Simultaneous detection of *Plasmodium vivax* and *Plasmodium falciparum* gametocytes in clinical isolates by multiplex-nested RT-PCR. Malar J.

[31] Miller RH, Obuya CO, Wanja EW, Ogutu B, Waitumbi J, Luckhart S (2015). Characterization of *Plasmodium ovale curtisi* and *P. ovale wallikeri* in Western Kenya utilizing a novel species-specific real-time PCR assay. PLoS Negl Trop Dis.

[32] Schneider P, Reece SE, van Schaijk BC, Bousema T, Lanke KH, Meaden CS (2015). Quantification of female and male *Plasmodium falciparum* gametocytes by reverse transcriptase quantitative PCR. Mol Biochem Parasitol.

[33] Briand V, Le Hesran JY, Mayxay M, Newton PN, Bertin G, Houze S (2016). Prevalence of malaria in pregnancy in southern Laos: a cross-sectional survey. Malar J.

[34] Lennon SE, Miranda A, Henao J, Vallejo AF, Perez J, Alvarez A (2016). Malaria elimination challenges in Mesoamerica: evidence of submicroscopic malaria reservoirs in Guatemala. Malar J.

[35] Imwong M, Nguyen TN, Tripura R, Peto TJ, Lee SJ, Lwin KM (2015). The epidemiology of subclinical malaria infections in south–east Asia: findings from cross-sectional surveys in Thailand–Myanmar border areas, Cambodia, and Vietnam. Malar J.

[36] Cheng Z, Wang D, Tian X, Sun Y, Sun X, Xiao N (2015). Capture and ligation probe-PCR (CLIP-PCR) for molecular screening, with application to active malaria surveillance for elimination. Clin Chem.

[37] Wang Q, Zhao Z, Zhang X, Li X, Zhu M, Li P (2016). Naturally acquired antibody responses to *Plasmodium vivax* and *Plasmodium falciparum* Merozoite Surface Protein 1 (MSP1) C-Terminal 19 kDa domains in an area of unstable malaria transmission in Southeast Asia. PLoS ONE.

[38] Laman M, Moore BR, Benjamin J, Padapu N, Tarongka N, Siba P (2014). Comparison of an assumed versus measured leucocyte count in parasite density calculations in Papua New Guinean children with uncomplicated malaria. Malar J.

[39] Snounou G, Singh B (2002). Nested PCR analysis of Plasmodium parasites. Methods Mol Med.

[40] Yan J, Li N, Wei X, Li P, Zhao Z, Wang L (2013). Performance of two rapid diagnostic tests for malaria diagnosis at the China–Myanmar border area. Malar J.

[41] Yin J, Xia Z, Yan H, Huang Y, Lu L, Geng Y (2013). Verification of clinically diagnosed cases during malaria elimination programme in Guizhou Province of China. Malar J.

[42] Zhu X, Zhao Z, Feng Y, Li P, Liu F, Liu J (2016). Genetic diversity of the *Plasmodium falciparum* apical membrane antigen I gene in parasite population from the China–Myanmar border area. Infect Genet Evol.

[43] Wampfler R, Mwingira F, Javati S, Robinson L, Betuela I, Siba P (2013). Strategies for detection of Plasmodium species gametocytes. PLoS ONE.

[44] Wu L, van den Hoogen LL, Slater H, Walker PG, Ghani AC, Drakeley CJ (2015). Comparison of diagnostics for the detection of asymptomatic *Plasmodium falciparum* infections to inform control and elimination strategies. Nature.

[45] Poschl B, Waneesorn J, Thekisoe O, Chutipongvivate S, Karanis P (2010). Comparative diagnosis of malaria infections by microscopy, nested PCR, and LAMP in northern Thailand. Am J Trop Med Hyg.

[46] Anthony C, Mahmud R, Lau YL, Syedomar SF, La Sri Sri, Ponnampalavanar S (2013). Comparison of two nested PCR methods for the detection of human malaria. Trop Biomed.

[47] Alemayehu S, Feghali KC, Cowden J, Komisar J, Ockenhouse CF, Kamau E (2013). Comparative evaluation of published real-time PCR assays for the detection of malaria following MIQE guidelines. Malar J.

[48] McNamara DT, Kasehagen LJ, Grimberg BT, Cole-Tobian J, Collins WE, Zimmerman PA (2006). Diagnosing infection levels of four human malaria parasite species by a polymerase chain reaction/ligase detection reaction fluorescent microsphere-based assay. Am J Trop Med Hyg.

[49] Yarosh HL, Hyatt CJ, Meda SA, Jiantonio-Kelly R, Potenza MN, Assaf M (2014). Relationships between reward sensitivity, risk-taking and family history of alcoholism during an interactive competitive fMRI task. PLoS ONE.

[50] Plowe CV, Djimde A, Bouare M, Doumbo O, Wellems TE (1995). Pyrimethamine and proguanil resistance-conferring mutations in *Plasmodium falciparum* dihydrofolate reductase: polymerase chain reaction methods for surveillance in Africa. Am J Trop Med Hyg.

[51] Murphy SC, Prentice JL, Williamson K, Wallis CK, Fang FC, Fried M (2012). Real-time quantitative reverse transcription PCR for monitoring of blood-stage *Plasmodium falciparum* infections in malaria human challenge trials. Am J Trop Med Hyg.

[52] Zimmerman PA (2015). Nucleic Acid surveillance and malaria elimination. Clin Chem.

[53] Bousema T, Okell L, Shekalaghe S, Griffin JT, Omar S, Sawa P (2010). Revisiting the circulation time of *Plasmodium falciparum* gametocytes: molecular detection methods to estimate the duration of gametocyte carriage and the effect of gametocytocidal drugs. Malar J.

