# Supplementary material for: Comparison of methods for detecting asymptomatic malaria infections in the China–Myanmar border area
Source: Malar J. 2017 Apr 20;16:159. doi: 10.1186/s12936-017-1813-0 (PMC5397696; doi:10.1186/s12936-017-1813-0)
Supplement: Supplementary file 1 — Additional file 1. PCR primer sequences and reaction conditions. [file 12936_2017_1813_MOESM1_ESM.doc]

**Additional file 1. PCR p**rimer sequences and reaction conditions

| Gene | Primer name | Primer sequence | PCR conditions | Product size (bp) | References |
| --- | --- | --- | --- | --- | --- |
| *Plasmodium sp.* | rPLU1  rPLU5 | TCAAAGATTAAGCCATGCAAGTGA  CCTGTTGTTGCCTTAAACTTC | First PCR: 95°C for 5 min; 94°C for 30 s, 55°C for 1 min, 72°C for 1 min, 35 cycles; 72°C 5 min. | -1670 | [12, 27,39] |
| *P.falciparum* | rFAL1  rFAL2 | TTAAACTGGTTTGGGAAAACCAAATATATT  ACACAATGAACTCAATCATGACTACCCGTC | Nested PCR: 95°C for 5 min; 94°C for 40 s, 58°C for 1min, 72°C for 2 min, 30 cycles; 72°C 5min. | 205 | [12, 31, 39，40] |
| *P. vivax* | PV18S F  PV18S R | GAATTTTCTCTTCGGAGTTTATTC  GTAGAAAAGGGAAAGGGAAACTGTTA | Nested PCR: 95°C for 5 min; 94°C for 40 s, 58°C for 1 min, 72°C for 2 min, 30 cycles; 72°C 5 min. | 419 | [12, 39，40] |
| *P.malariae* | rMAL1  rMAL2 | ATAACATAGTTGTACGTTAAGAATAACCGC  AAAATTCCCATGCATAAAAAATTATACAAA | Nested PCR: 95°C for 5 min; 94°C for 1 min; 58°C for 1 min; 72°C for 2 min, 30 cycles; 72°C 5 min | 145 | [27, 31, 39] |
| *P. ovale* | rOVA1WC  rOVA2WC | TGTAGTATTCAAACGCAGT  TATGTACTTGTTAAGCCTTT | Nested PCR: 95°C for 5 min; 94°C for 1 min; 58°C for 1 min; 72°C for 2 min, 30 cycles; 72°C 5 min | 659-662 | [27, 31, 39] |
| *P. vivax* | pvs25F  pvs25R | CACTTAGCCAAAATGAACTC  AAAGGACAAGCAGGATGATA | PCR: 94°C for 2 min; 94°C for 15 s; 54°C for 15 s; 68°C for 1 min, 35 cycles; 68°C 5 min | 645 | [42] |
| *P. vivax* | pvs25_fw pvs25_rev | ACACTTGTGTGCTTGATGTATGTC  ACTTTGCCAATAGCACATGAGCAA | PCR: 94^o^C for 1 min; 94 ^o^C for 40 s, 53 ^o^C for 30 s, 72 ^o^C for 30 s, 35 cycles; 72 ^o^C 5 min. | 115 | [43] |
|  | pvs25_fw pvs25_rev  pvs25_probe | ACACTTGTGTGCTTGATGTATGTC  ACTTTGCCAATAGCACATGAGCAA  FAM-TGCATTGTTGAGTACCTCTCGGAA-BHQ1 | qPCR: 95°C for 30 s; 95°C for 5 s, 60°C for 34 s, 40 cycles | 115 | [43] |
