# Supplementary figures and images for: Comparison of methods for detecting asymptomatic malaria infections in the China–Myanmar border area
Source: Malar J. 2017 Apr 20;16:159. doi: 10.1186/s12936-017-1813-0 (PMC5397696; doi:10.1186/s12936-017-1813-0)

## Slide 1
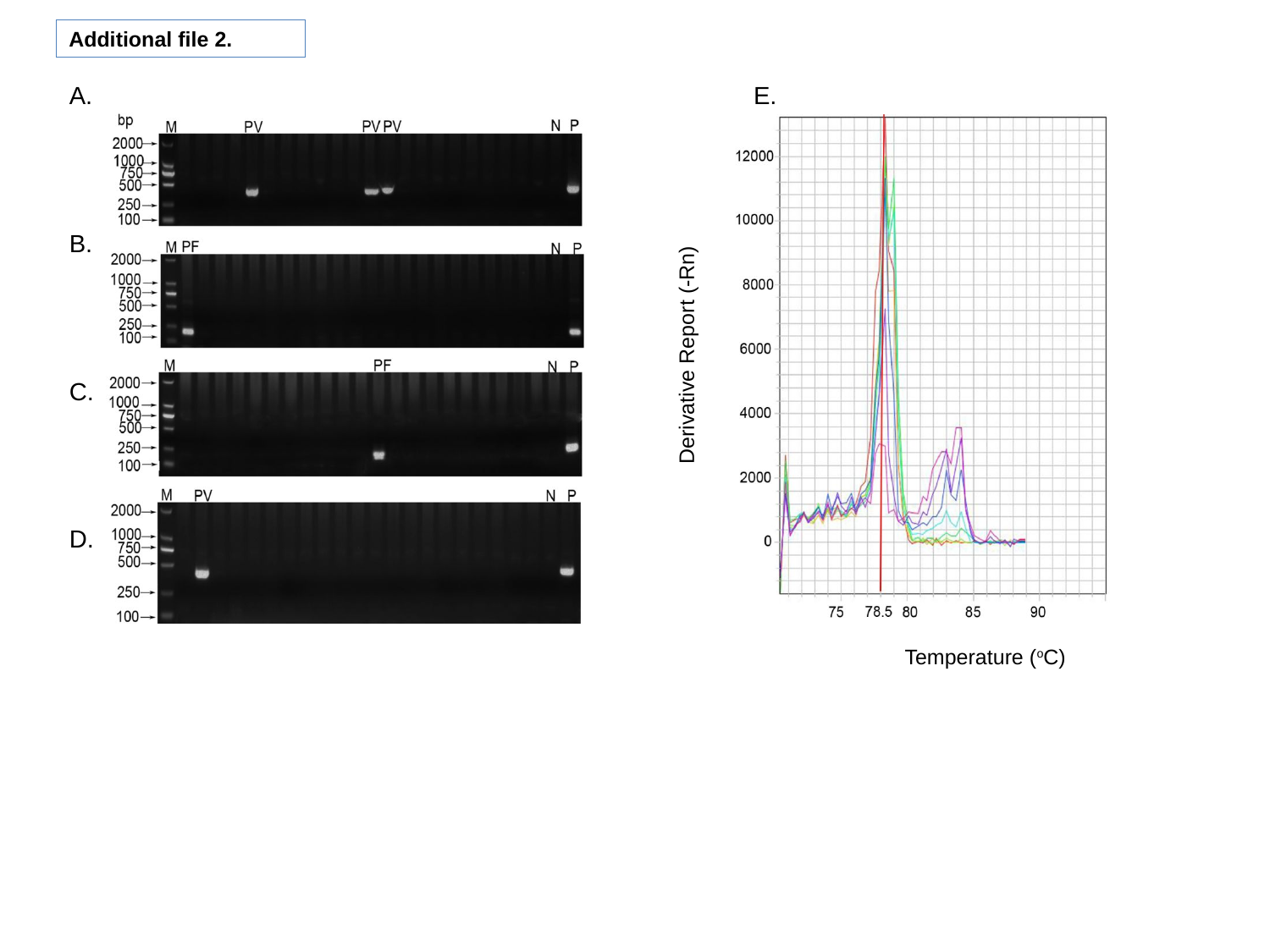

Additional file 2.
A.
B.
C.
D.
E.
Derivative Report (-Rn)
Temperature (oC)

Supplement: Supplementary file 2 — Additional file 2. Nested PCR targeting 18S rRNA with parasite genomic DNA (nD-PCR) and cDNA (nRT-PCR). A. PV-18S rRNA (419 bp) by nD-PCR. B. PF-18S rRNA (205 bp) by nD-PCR. C. PF-18S rRNA (205 bp) by nRT-PCR. D. PV-18S rRNA (419 bp) by nRT-PCR. E. CLIP-PCR positive sample definedby normal “S” amplification, the dissolution curve has a single peak, and the product Tm is the same as that in positive control (with <0.5 °C difference). PCR products were separated on 1.2% agarose gels (A-D). M, molecular markers in bp. N = negative control, P = positive control. [file 12936_2017_1813_MOESM2_ESM.pptx]

## Slide 1
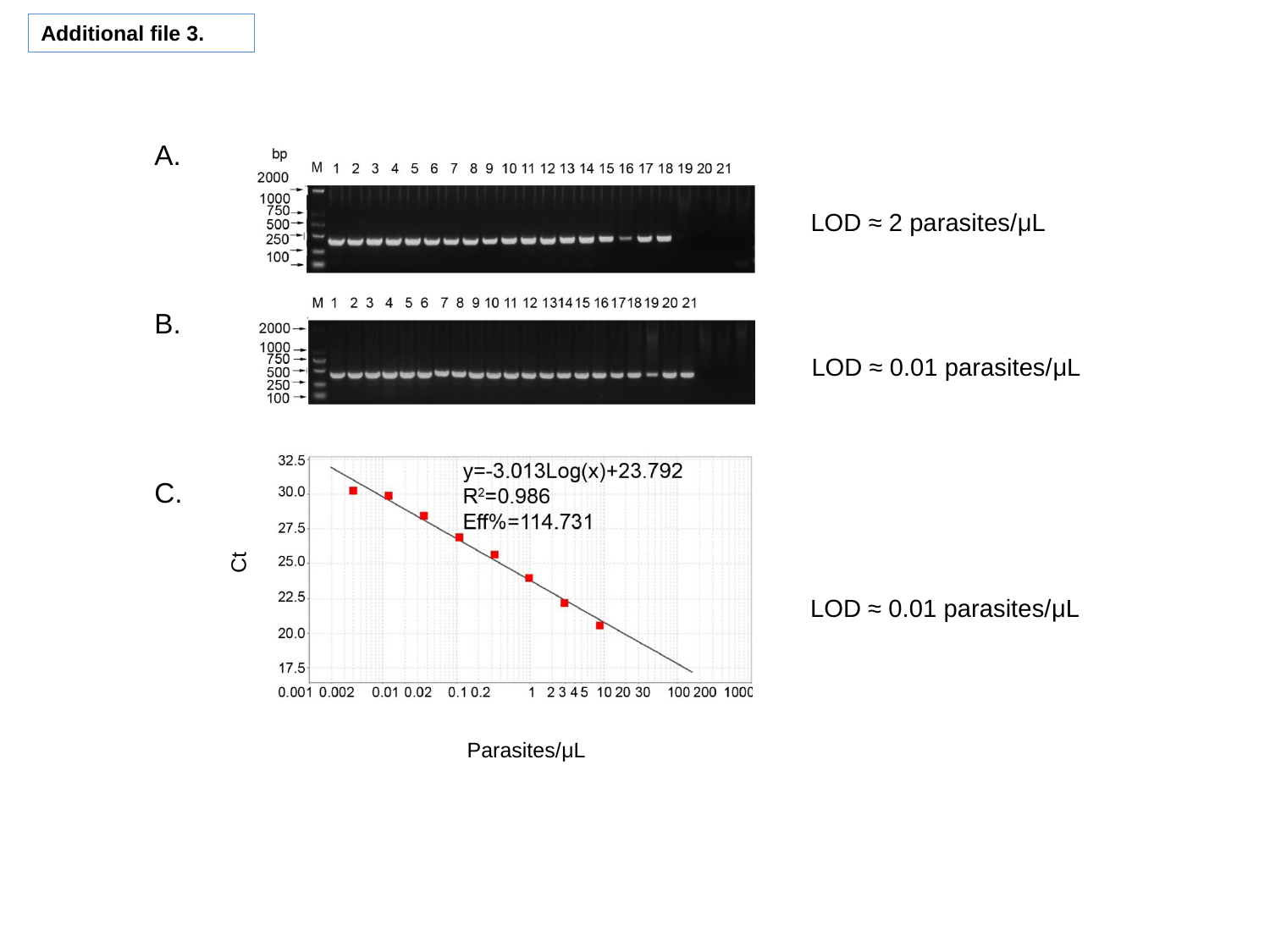

Additional file 3.
A.
B.
C.
LOD ≈ 2 parasites/μL
LOD ≈ 0.01 parasites/μL
Ct
LOD ≈ 0.01 parasites/μL
Parasites/μL

Supplement: Supplementary file 3 — Additional file 3. Limits of detection (LOD) of different detection methods - nD-PCR, nRT-PCR and CLIP-PCR. A. LOD based on P. vivax 18S rRNA gene by nD-PCR. Four-fold serial dilutions of the parasites from 2000 to 0.488 parasites/μL were used. Lanes 1-3, 4-6, 7-9, 10-12, 13-15, 16-18 and 19-21 correspond to parasite density of 2000, 500, 125, 31.25, 7.81, 1.95, 0.49 parasites/μL, respectively. B. LOD based on P. vivax 18S rRNA by nRT-PCR. Three-fold serial dilutions of the parasites from 5.6 to 0.0026 parasites/μL were used. Lanes 1-3, 4-6, 7-9, 10-12, 13-15, 16-18, 19-21 and 22-24 represent 5.6, 1.87, 0.533, 0.178, 0.059, 0.02, 0.0078 and 0.0026 parasites/μL, respectively. C. LOD of CLIP-PCR. Three-fold serial dilutions of P. falciparum 3D7 were used (8 – 0.004 parasites/μL). Eff% is amplification efficiency. Ct values from duplicate tests were plotted against parasite densities. [file 12936_2017_1813_MOESM3_ESM.pptx]
