# Supplementary material for: Comparison of methods for detecting asymptomatic malaria infections in the China–Myanmar border area
Source: Malar J. 2017 Apr 20;16:159. doi: 10.1186/s12936-017-1813-0 (PMC5397696; doi:10.1186/s12936-017-1813-0)
Supplement: Supplementary file 4 — Additional file 4. Limits of detection (LOD) of RT-PCR and qRT-PCR for gametocyte detection. A. LOD based on P. vivax Pvs25 rRNA gene by RT-PCR. Three-fold serial dilutions of the gametocytes (5.6 – 0.0026 gametocytes/μL) were used. Lanes 1-3, 4-6, 7-9, 10-12, 13-15, 16-18, 19-21 and 22-24 correspond to gametocyte density of 5.6, 1.87, 0.533, 0.178, 0.059, 0.02, 0.0078 and 0.0026 gametocytes/μL, respectively. B. LOD of qRT- PCR using plasmid DNA. Ten-fold serial dilutions of P. vivax Pvs25 plasmid were used (3 ×10^9^ – 0.3 copies/μL). Eff% is the amplification efficiency. Ct values from duplicate tests were plotted against parasite densities. C. LOD of qRT-PCR using P. vivax gametocytes. Three-fold serial dilutions of P. vivax gametocytes (9 – 0.001 gametocytes/μL) in three samples were used. Eff% is the amplification efficiency. Ct values from duplicate tests were plotted against parasite densities. [file 12936_2017_1813_MOESM4_ESM.pptx]

## Slide 1
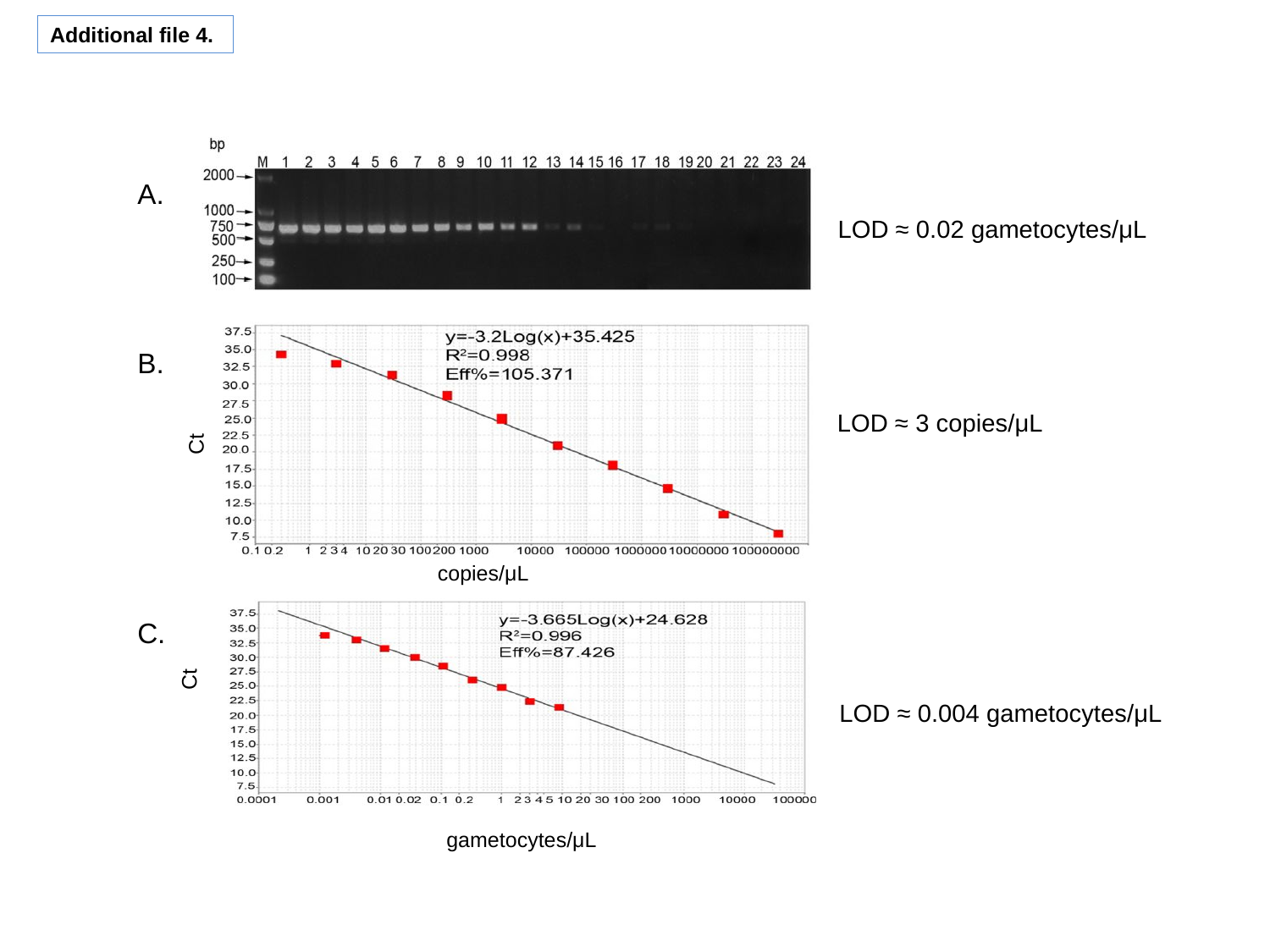

Additional file 4.
A.
B.
C.
Ct
gametocytes/μL
LOD ≈ 0.02 gametocytes/μL
LOD ≈ 3 copies/μL
copies/μL
Ct
LOD ≈ 0.004 gametocytes/μL
